# Protein-specific immune response elicited by the *Shigella sonnei* 1790GAHB GMMA-based candidate vaccine in adults with varying exposure to *Shigella*

**DOI:** 10.1128/msphere.01057-24

**Published:** 2025-04-16

**Authors:** Arlo Z. Randall, Valentino Conti, Usman Nakakana, Xiaowu Liang, Andy A. Teng, Antonio Lorenzo Di Pasquale, Melissa Kapulu, Robert Frenck, Odile Launay, Pietro Ferruzzi, Antonella Silvia Sciré, Elisa Marchetti, Christina Obiero, Jozelyn V. Pablo, Joshua Edgar, Philip Bejon, Adam D. Shandling, Joseph J. Campo, Angela Yee, Laura B. Martin, Audino Podda, Francesca Micoli

**Affiliations:** 1 Antigen Discovery, Inc. (ADI), Irvine, California, USA; 2 GSK Vaccines Institute for Global Health 622628 , Siena, Italy; 3 Biosciences Department, Kenya Medical Research Institute (KEMRI)-Wellcome Trust Programme, Kilifi, Kenya; 4 Centre for Tropical Medicine and Global Health, Nuffield Department of Medicine, University of Oxford 6396, Oxford, United Kingdom; 5 Division of Infectious Diseases, Cincinnati Children’s Hospital Medical Center, Cincinnati, Ohio, USA; 6 Université Paris Cité; Assistance Publique Hôpitaux de Paris, CIC Cochin Pasteur; Inserm, Paris, France; 7 Clinical Research Department, Kenya Medical Research Institute (KEMRI)-Wellcome Trust Programme, Kilifi, Kenya; University of Wyoming College of Agriculture Life Sciences and Natural Resources, Laramie, Wyoming, USA

**Keywords:** protein microarray, proteome immune profiling, *Shigella*, vaccine, Generalized Modules for Membrane Antigens (GMMA)

## Abstract

*Shigella* is a leading cause of diarrheal morbidity and mortality in young children from low- and middle-income countries. Here, we aimed to verify the ability of the generalized modules for membrane antigens (GMMA)-based *Shigella sonnei* candidate vaccine 1790GAHB to elicit an anti-protein antibody response. Serum samples from previous clinical trials in adults (a dose-escalation study and its extension in France, a vaccine efficacy study after human challenge in the United States, and a study in Kenya) were investigated using pan-proteome microarrays consisting of 3,150 full-length or fragmented *Shigella* proteins. Pre-/post-vaccination comparisons identified subsets of proteins that were highly immunoreactive and largely overlapped across all trials; the T3SS lipochaperone family protein (expressed on GMMA) was the most reactive in all studies. Responses to several microarray antigens correlated well with *S. sonnei* LPS serum IgG antibody levels. Overall, we confirmed the ability of GMMA to elicit an anti-protein IgG/IgA response; however, no association with protection against shigellosis was identified. In the challenge study, IgG response to seven antigens (IpaC, IpaB, IpaA, IpaD, IpaH, IpgC, and MxiD; not expressed on GMMA) was associated with a decreased risk of shigellosis. These antigens were observed to also have high IgG responses at baseline in individuals naturally exposed to *Shigella* and could constitute targets for future vaccine development.

**IMPORTANCE:**

*Shigella* remains a major cause of diarrheal disease, especially in children aged under 5 years from low-to-middle-income countries. No vaccine against shigellosis is yet widely available despite the high public health need. An ideal vaccine would provide protection against the most prevalent species, *Shigella flexneri* and *Shigella sonnei*; therefore, it could be relevant to identify common antigens. We developed a microarray containing 3,150 full-length or fragmented proteins selected across *Shigella* species. Sera collected in four clinical trials conducted in three countries of varying endemicity to evaluate a *S. sonnei* GMMA-based candidate vaccine were tested against these proteins. We identified several *Shigella* proteins (IpaC, IpaB, IpaA, IpaD, IpaH, IpgC, MxiD) that induced robust antibody response following experimental challenge or natural infection. These proteins correlated with a reduced risk of shigellosis after the *S. sonnei* challenge. We found no apparent role for anti-GMMA proteins’ IgG or IgA response in protection against shigellosis.

## INTRODUCTION

*Shigella* spp. are bacterial enteric pathogens that spread via the fecal–oral route and lead to a variety of symptoms ranging from mild, self-limiting diarrhea to severe bacillary dysentery (1, 2). Worldwide, *Shigella* is one of the leading causes of diarrheal morbidity and mortality, particularly in children younger than 5 years living in low- and middle-income countries (LMICs), where it is estimated that nearly 100,000 children per year die from the infection (1, 3–5). Other vulnerable populations include people with immune deficiency disorders, men who have sex with men, and international travelers to shigellosis-endemic countries (1). Over the past few decades, the indiscriminate use of antibiotics has played an important role in the emergence of multidrug-resistant *Shigella* variants (6–8), worsening the control of shigellosis worldwide.

Four *Shigella* species have been identified, but epidemiological data indicate that *S. flexneri* (with more than 15 serotypes and sub-serotypes) and *S. sonnei* are responsible for almost 90% of shigellosis worldwide (1, 9, 10). *S. flexneri* is the most predominant in LMICs, while *S. sonnei* is prevalent in high-income countries (1, 7, 10, 11). However, the emergence of *S. sonnei* in the detriment of *S. flexneri* has been observed in the last decade in LMICs as well, in particular in industrialized areas (12, 13).

The development of a safe and efficacious vaccine would enhance the control of shigellosis. However, to date, no licensed vaccine against *Shigella* is widely available (14–16). The immune response against *Shigella* is believed to be serotype-specific (17–19) and mediated by the O antigen (OAg), which is part of the *Shigella* lipopolysaccharide (LPS) (15). Therefore, OAg has been the main target of vaccines under development. While the OAg structure and composition differ between serotypes and sub-serotypes, there is growing evidence that immunoglobulin (Ig) G and IgA antibodies against OAg are associated with immunity—albeit serotype-dependent—following both natural (20) or experimental exposure (challenge) in controlled human infection model (CHIM) studies (21–24). However, additional potential immunological targets are invasion plasmid antigen (Ipa) proteins and virulence (Vir) factors (14), which are part of the machinery by which *Shigella* infects host cells. IpaB and IpaC (both type III secretion system [T3SS] proteins) and the autotransporter protein IcsA (or VirG) have also been explored as potential targets of serotype-independent subunit vaccines against shigellosis due to their functional roles during *Shigella* infection and pathogenesis (14, 20, 25, 26). Despite the considerable effort dedicated to vaccine development, the immunological mechanisms of protection against shigellosis are not yet fully defined. Therefore, to design a vaccine able to induce cross-reactive immune responses, it can be important to identify other *Shigella*-specific antigens that are common to all serotypes and that correlate with immunity and protection from the disease.

The 1790GAHB candidate vaccine is a generalized modules for membrane antigens (GMMA)-based vaccine (27). Its development represented an important stepping stone for the design of an improved version of a four-component vaccine (altSonflex1-2-3) formulated against three additional *Shigella* serotypes (*S. flexneri* 1b, 2a, and 3a) (28). altSonflex1-2-3 was shown to induce functional immune responses against all four serotypes in healthy European adults without safety signals or concerns (29). The GMMA have been proposed as a delivery system for the OAg, but they also present multiple proteins to the immune system (30, 31).

**Fig 1:**
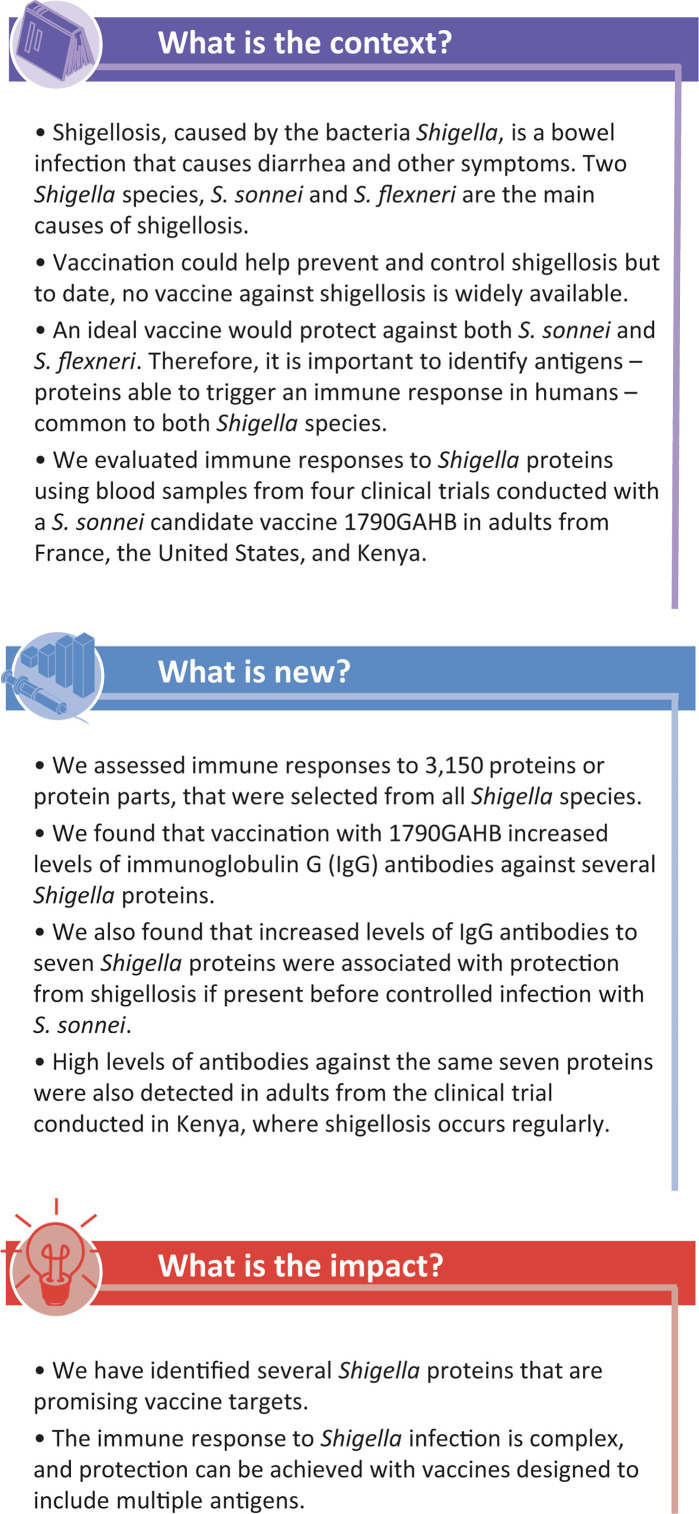
Plain language summary.

Over the last years, protein microarrays have been used successfully to profile immune responses following natural exposure, challenge, or vaccination and to identify immune signatures predictive of protection against various diseases, such as *Shigella*, malaria, or meningitis B (22, 32–34). The aim of the current study was to evaluate the ability of the GMMA-based vaccine to elicit an anti-protein response utilizing adult serum samples from participants from studies conducted in endemic and non-endemic areas and participants in a CHIM study with the *S. sonnei* 53G strain. We profiled and characterized pre-existing responses in each setting, responses to vaccination in each setting, and correlation with protection against shigellosis post-challenge, as well as differences between settings. A summary contextualizing the results and potential clinical relevance and impact of the research is provided in the plain language summary (Fig. 1).

## MATERIALS AND METHODS

### Clinical studies and serum samples

This proteomic analysis was conducted on selected serum samples derived from four clinical trials: H03_01TP (NCT02017899) and its extension H03_01E1TP (NCT03089879), H03_03TP (NCT03527173), and H03_04TP (NCT02676895). Details of the study designs and eligibility criteria were previously described (35–38).

**Fig 2:**
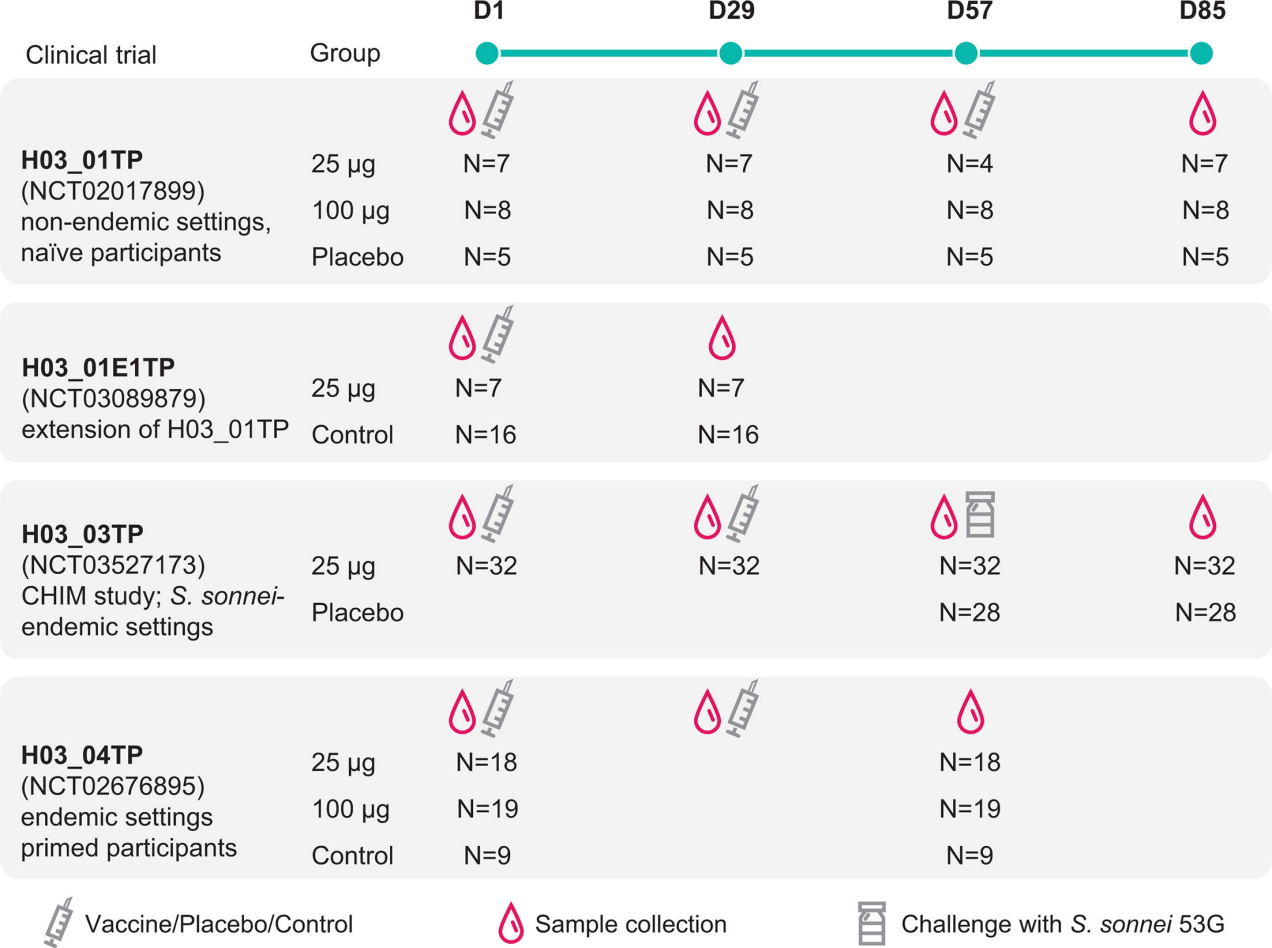
Overview of the study samples. Serum samples were collected from clinical trials evaluating the GMMA-based *S. sonnei* candidate vaccine 1790GAHB in adults with various exposures to *Shigella* and tested for protein-specific antibody response using a pan-proteome microarray. The syringes indicate vaccination with 1790GAHB in vaccine groups (25 µg and 100 µg groups) and placebo/control vaccine administration in placebo/control groups, while the blood drops indicate the timing of blood samples. CHIM, controlled human infection model; D, day; N, number of samples. Note: in the H03_01E1TP study, participants in the 25 µg group had received primary vaccination in the H03_01TP study 2–3 years earlier.

The serum samples used in the current study were obtained from each clinical trial and collected at the time points indicated in Fig. 2, generally at baseline (pre-vaccination) and post-each vaccination and pre- and post-challenge for samples from the H03_03TP study. Samples were selected according to availability and the participants’ dose group to allow relevant comparison. A total of 399 serum samples from 149 participants in the four clinical trials were tested from groups receiving 1790GAHB at dosages of 1.5/25 (25 µg group) and 5.9/100 (100 µg group) µg of OAg/µg of protein and placebo/control when available. Paired samples (collected from the same individual) were used for comparisons between different time points.

Briefly, the H03_01TP study was a phase 1, dose-escalation trial conducted in France between February 2014 and March 2015 to evaluate the safety and immunogenicity of the 1790GAHB vaccine in healthy adults. Participants received three injections of the 1790GAHB vaccine (containing 0.059/1, 0.29/5, 1.5/25, 2.9/50, or 5.9/100 µg of OAg/µg of protein) or placebo, and serum samples were taken at baseline and 28 days after each dose (35). In the H03_01E1TP extension trial conducted between March 2017 and August 2017, all eligible participants from H03_01TP received a single injection of 1790GAHB containing 1.5/25 µg of OAg/µg of protein at 2–3 years after primary vaccination (36, 39).

The H03_03TP study was a phase 2b human challenge trial conducted in the United States between August 2018 and November 2019 to evaluate the safety, immunogenicity, and efficacy of 1790GAHB. Healthy adults were randomized to receive either two injections of the 1790GAHB vaccine (1.5/25 µg of OAg/µg of protein) or placebo, followed by an oral challenge dose of the *S. sonnei* 53G strain, 28 days after their second vaccination (37).

The H03_04TP study was a phase 2a controlled trial conducted in Kenya between August 2016 and March 2017 to evaluate the safety and immunogenicity of 1790GAHB in healthy adults in a shigellosis-endemic area. Participants received two injections of 1790GAHB at one of two dosages: 1.5/25 and 5.9/100 µg of OAg/µg of protein. Controls received vaccines against meningococcus (first vaccination) and tetanus, diphtheria, and acellular pertussis (second vaccination) (38).

### Protein microarray construction

*Shigella* pan-proteome microarrays were produced by Antigen Discovery, Inc. (Irvine, CA, USA). A clone library was constructed targeting 3,150 full-length or fragmented proteins selected to represent the core proteome and species-specific proteins from *S. boydii* (*n* = 2,040), *S. dysenteriae* (*n* = 250), *S. flexneri* (*n* = 460), and *S. sonnei* (*n* = 400). Clones were sequenced (Retrogen, Inc., San Diego, CA, USA), and the results matched the correct target for all 3,150 clones. Additional information on the protein microarray construction can be found in the supplemental material. Details for each array spot (i.e., array spot identifier, *Shigella* species, protein description, and amino acid sequence) are given in Table S1.

### Probing

From each clone, the corresponding protein was expressed using a coupled *Escherichia coli*-based *in vitro* transcription and translation (IVTT) system. The expressed proteins were printed onto nitrocellulose-coated glass slides. Each expressed protein included a 5′-polyhistidine and a 3′-hemagglutinin epitope tag, and microarray protein expression and chip printing were quality checked using anti-histidine and anti-hemagglutinin monoclonal antibodies with fluorescently labeled secondary antibodies. Serum samples were diluted 1:100 in a 1.5 mg/mL *E. coli* lysate solution (BiotechRabbit, Berlin, Germany) in protein arraying buffer (Maine Manufacturing, Sanford, ME, USA) and incubated at room temperature for 30 min. Arrays were then rehydrated in blocking buffer for 30 min. After removing the blocking buffer, arrays were probed with diluted serum samples using sealed, fitted slide chambers to avoid cross-contamination between arrays. The arrays were incubated overnight at 4°C with agitation, washed three times with tris-buffered saline (TBS)−0.05% Tween 20, and incubated with either biotin anti-human IgA (1:500) or Cy3 goat anti-human IgG (1:200, both from Jackson ImmunoResearch, West Grove, PA, USA) at room temperature. Arrays were washed three times with TBS-0.05% Tween 20. The samples incubated with biotin anti-human IgA were then incubated with streptavidin-conjugated SureLight P-3 (Columbia Biosciences, Frederick, MD, USA) at room temperature, followed by three more washes with TBS-0.05% Tween, protected from light. Following IgA incubations, the samples were incubated with Cy3 donkey anti-human IgG (1:200, Jackson ImmunoResearch) or DyLight 650 goat anti-human IgG (1:250, Bethyl Laboratories, Montgomery, TX, USA) at room temperature. After washing another six times with TBS–0.05% Tween 20 and once with water, arrays were air dried by centrifugation at 400 *g* for 7 min and left overnight in a desiccator before scanning.

### Baseline dilution experiments

To confirm the selection of 1:100 for the dilution of serum samples, baseline dilution experiments were performed (Table S2). Baseline samples from the H03_04TP trial conducted in Kenya, a *Shigella*-endemic setting, were used to make two random pools containing baseline samples from probing days 1 (pool 1, *n* = 24) and 2 (pool 2, *n* = 24). Both pools were probed at the following dilutions: 1:100, 1:200, 1:400, 1:800, 1:1,600, and 1:3,200. Data obtained from the probing of the pools followed the expected proportional decrease in raw signal for the IVTT control spots and most IVTT antigen spots. However, for the most reactive spots, the raw signal dropped much less than expected, indicating binding saturation is occurring at 1:100 dilution and, to a lesser degree, at all the dilutions up to 1:1,600. Data for the most reactive proteins on the array obtained at 1:100 and 1:1,600 dilutions are shown in Table S2. These results supported the selection of the 1:100 dilution used in all experiments. All experiments were performed by Antigen Discovery, Inc.

### Microarray signal quantification

The microarray slides were scanned using a GenePix 4300A high-resolution microarray scanner (Molecular Devices, Sunnyvale, CA, USA), and auto-gridding software (Mapix, Innopsys, Chicago, IL, USA) was used to define the array and sub-array layout. For each spot on the slide, the foreground intensity (median of pixels inside the circle defining the spot) and the local background intensity (median of pixels just outside the circle defining the spot) were quantified, and the final raw intensity value was calculated as the foreground intensity minus the local background intensity. Log-2-transformed raw signal values were normalized to remove systematic effects by subtracting the median signal intensity of the IVTT control spots (not expressing proteins) for each sample. Since the IVTT control spots carry not only the chip, sample, and batch-level systematic effects, but also antibody background reactivity to the IVTT system, this procedure normalizes the data and provides a relative measure of the specific versus the nonspecific antibody binding to the IVTT controls. Thus, a normalized result of 0.0 indicates no difference versus the IVTT controls, while a value of 1.0 indicates a doubling with respect to the IVTT controls.

### Statistical analysis

For statistical analysis and visualization, reactivity filtering was performed by defining seropositivity as a normalized signal of 1.0 or greater. Spots were categorized as reactive and carried forward for statistical analysis if at least 10% of samples were seropositive in one or more of the participant groups (i.e., each combination of sample day and treatment groups).

The following metrics were calculated for each reactive array spot individually: group means, group mean differences, counts of samples over the reactivity threshold in each group, raw and false discovery corrected *P* values from *t*-test and non-parametric Wilcoxon rank test, and the area under the receiver operating characteristics curve. For paired comparisons, *t*-test *P* values were calculated as paired tests. Multiple test correction was performed using the Benjamini–Hochberg method for controlling the false discovery rate (40). The number of participants with at least a 50% increase in signal from day 1 (considered an expression of high immunoreaction) were calculated as an additional metric when relevant.

Associations between increases from baseline for *S. sonnei* LPS serum IgG levels (base-2 log of anti-LPS titers) as previously determined by enzyme-linked immunosorbent assay (ELISA) and the normalized intensities of array spots (IgG or IgA data) were evaluated using the Spearman rank correlation coefficient.

All data processing, statistical analysis, and visualizations were performed by Antigen Discovery, Inc using R (http://www.R-project.org).

## RESULTS

**Fig 3:**
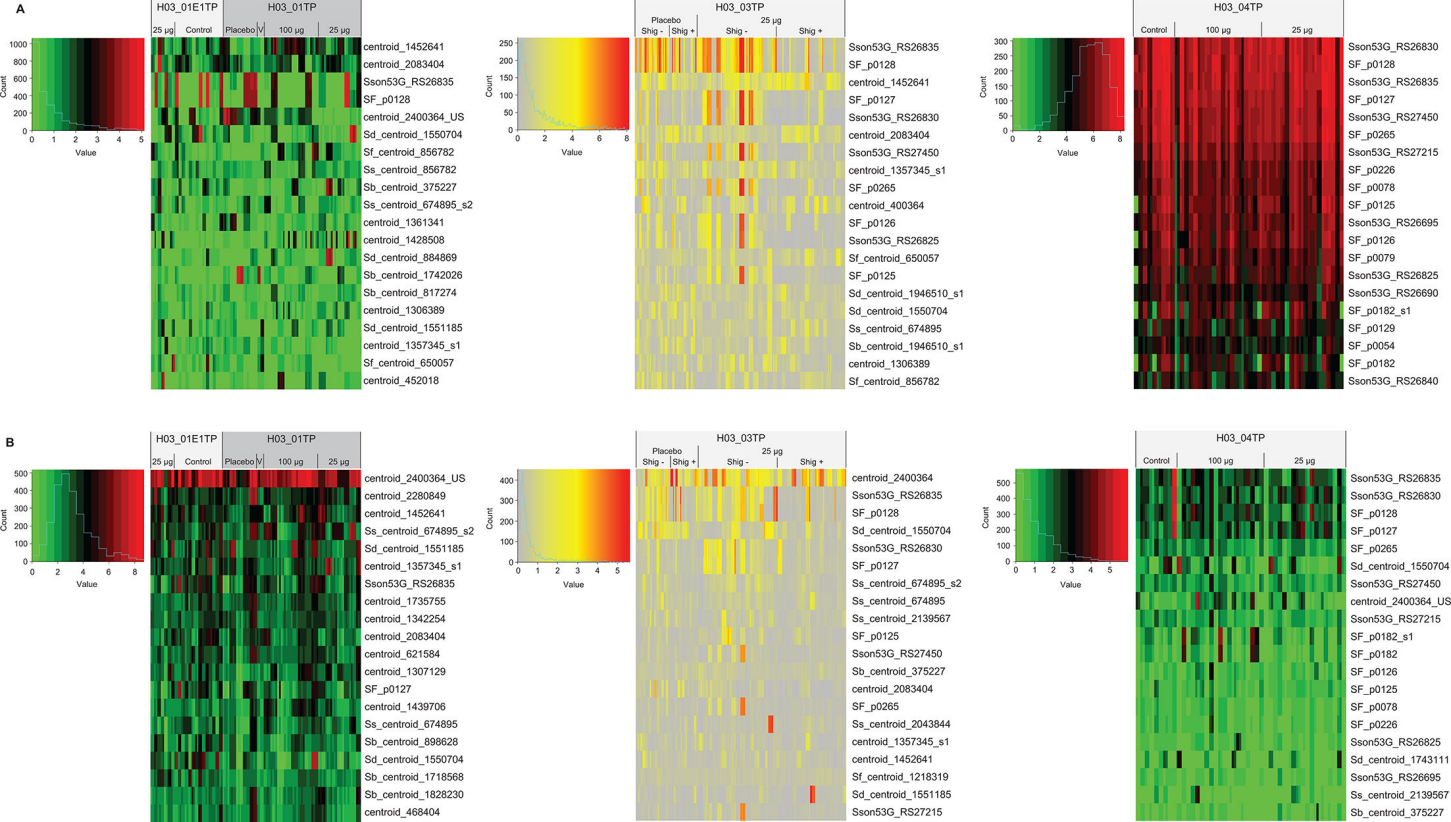
*Shigella*-specific IgG (**A**) and IgA (**B**) responses to immunogenic proteins from the serum samples of healthy adults from four clinical trials of 1790GAHB. Heatmaps of normalized IgG and IgA signal intensities of the 20 most reactive proteins using all available serum samples measured on a *Shigella* pan-proteome microarray are presented for each clinical trial. The color key and histograms are shown for each heatmap. Antigens are ranked by average reactivity. The array spot identifier is given; a full description for each spot is provided in Table S1. For the H03_03TP study, participants were grouped in individuals without shigellosis (Shig−) or with shigellosis (Shig+) following the challenge with the *S. sonnei* 53G strain.

To identify potentially immunogenic protein antigens following vaccination in all clinical trials, samples collected pre- and post-vaccination were assayed. Anti-protein IgG and IgA responses to the 20 most reactive proteins in the microarray ranked by average reactivity using all available samples in each of the four clinical trials are presented in Fig. 3.

### Antigens identified following vaccination with 1790GAHB

When testing serum samples collected in the H03_01TP trial (France, non-endemic setting) and its extension, paired comparisons between day 1 and post-vaccination days of all samples showed increases in *Shigella* protein antigen-specific IgG levels over time in most adults vaccinated with 1790GAHB, with the peak of the response observed at day 85 (28 days after the third administration). Statistically significant increases in IgG responses were observed for seven to 11 array spots using three different metrics. Five array spots were identified by all three metrics as having a significant increase post-vaccination, corresponding to the following proteins: T3SS lipochaperone family protein, putative outer membrane lipoprotein Wza, conserved hypothetical protein, outer membrane protein slp, and major outer membrane lipoprotein Lpp. After the booster vaccination in the H03_01E1TP study, a further ≥50% increase in IgG responses from baseline was seen in multiple participants for putative outer membrane lipoprotein Wza (in 4/7 participants; *t*-test *P* value = 0.02), T3SS lipochaperone family protein (3/7; *P* = 0.05), peptidoglycan-associated lipoprotein (2/7; *P* = 0.04), and outer membrane protein slp (2/7; *P* = 0.11). For IgA responses, an increase of ≥50% in ≥4 participants was only observed from baseline to day 29 in study H03_01E1TP for putative outer membrane lipoprotein Wza.

When results were stratified by study groups in the H03_01TP study, participants in the 100 µg group had more frequent and higher magnitude responses than those in the 25 µg group (Table S3). Of note, a direct comparison between dose groups was not statistically significant after correcting for multiple comparisons most likely due to the small sample size. For 12 array spots (which included the five identified as having a significant increase post-vaccination), ≥4 vaccinated participants in the 100 µg group showed an increase in the response ≥50% at day 85 compared to day 1 (Table 1; Table S3). IgG responses among participants receiving placebo were stable across all timepoints for all array spots (Table S3).

**TABLE 1:** Overview of *Shigella*-specific IgG responses after vaccination with 1790GAHB at doses of 5.9/100 µg of OAg/µg of protein (H03_01TP and H03_04TP studies) and 1.5/25 µg of OAg/µg of protein (H03_03TP study) in three clinical trials^*a*^^,*b*^

Array spot	Description	H03_01TP		H03_03TP		H03_04TP	
		Average difference (D85–D1); *P* value	*n*	Average difference (D57–D1); *P* value	*n*	Average difference (D57–D1); *P* value	*n*
centroid_1004904	Rare lipoprotein A family protein					**0.10; 0.06**	**2**
centroid_1279199	Outer membrane protein assembly factor BamC	0.73; 0.04	4	**0.25; 0.005**	**7**	**0.29; 0.02**	**4**
centroid_1306389	Conserved hypothetical protein	1.11; 0.005	6	**0.33; 0.01**	**6**	0.15; 0.02	1
centroid_1428508^*c*^	T3SS lipochaperone family protein	*2.09; 0.0007*	*8*	**0.93; 0.0002**	**15**	**0.84; 0.0002**	**10**
centroid_1452641	Peptidoglycan-associated lipoprotein	0.97; 0.003	6	**0.40; 0.001**	**10**	**0.70; 0.0004**	**9**
centroid_1637890	Tol Pal system beta propeller repeat protein TolB	1.08; 0.007	7	0.11; 0.02	2	**0.29; 0.001**	**4**
centroid_223856	BON domain protein	0.41; 0.03	4	0.08; 0.1	2	**0.17; 0.03**	**2**
centroid_2340470	Protein YceI			**0.09; 0.1**	**4**		
centroid_274565	Conserved hypothetical protein	*0.76; 0.003*	*5*	0.07; 0.009	0	0.08; 0.05	0
centroid_282246	Outer membrane protein slp	*2.06; 0.004*	*6*	**0.46; 0.0001**	**10**	**0.48; 0.001**	**3**
centroid_464893	SmpA/OmlA family protein					**0.38; 0.007**	**4**
centroid_468404	Major outer membrane lipoprotein	*0.62; 0.006*	*5*	0.07; 0.08	0	0.02; 0.4	0
centroid_476366	Periplasmic serine endoprotease DegP	0.82; 0.01	5	0.08; 0.007	0	0.15; 0.0009	0
centroid_600138	LppC lipofamily protein			**0.29; 0.002**	**5**	**0.28; 0.04**	**3**
centroid_601829	Putative outer membrane lipoprotein Wza	*0.60; 0.0002*	*4*	**0.24; 0.000009**	**4**	**0.27; 0.0004**	**3**
Sd_centroid_11661	P2 phage tail completion R family protein	0.60; 0.004	4	0.00; 0.9	0	0.06; 0.1	0
Whole array		*0.04*	*92*	*−0.005*	*396*	*0.019*	*112*

^*a*^ D, day; *n*, number of participants with ≥50% increase in signal from D1 to the considered timepoint; T3SS, type III secretion system; BON, bacterial osmotically inducible protein Y and nodulation; SmpA, small protein A; OmlA, outer membrane lipoprotein A; lppC, cytoplasmic membrane lipoprotein.

^*b*^ The array spots shown are the ones on the array where participants had an increase in signal from baseline to the considered timepoint of ≥50% in one of the H03_01TP, H03_03TP, and H03_04TP trials. The 12 array spots identified in the H03_01TP study were used as a basis for comparison between the three trials. The array spots are arranged in alphabetical order. For study H03_01TP, values in italics indicate the array spots for the five proteins that were identified as immunogenic when using all samples from the study and its extension. Values in bold indicate array spots for which an increase of ≥50% in signal was observed for ≥four participants in study H03_03TP and ≥10% of participants in study H03_04TP. *P* values were obtained using a paired *t*-test.

^*c*^ This array spot was identified as the most reactive in all three clinical trials.

When evaluating serum samples from the H03_03TP study (United States, *S. sonnei*-endemic setting), a ≥50% increase in IgG levels from day 1 to day 57 in ≥4 participants vaccinated with 1790GAHB was observed for eight array spots, and for seven of these spots, the increases were statistically significant using paired *t*-test (Table 1). However, only the increases observed for the array spot corresponding to the putative outer membrane lipoprotein Wza remained significant after multiple test corrections. Six of these array spots were also identified for samples from the H03_01TP study. For IgA, increases ≥50% in antibody levels from day 1 to day 57 were observed in ≥4 participants for an array spot corresponding to hemagglutinin family protein/adhesin.

When comparing IgG responses at days 1 and 57 for the samples from the H03_04TP study (Kenya, *Shigella*-endemic setting), levels in the 100 µg group were overall higher than in the 25 µg group (Fig. 3A). A ≥50% increase in IgG levels in ≥2 participants in the 100 µg group was observed for 10 array spots. For nine of these, the increases were statistically significant using paired *t*-test (Table 1), but none remained significant after multiple test corrections. Seven of the 10 array spots were also identified when assaying samples from the H03_01TP study, and six were identified with samples from the H03_03TP study. The array spot corresponding to the T3SS lipochaperone family protein was identified as the most reactive array spot in all three studies (Table 1). A ≥50% increase in IgA levels in ≥2 participants was observed for the array spot corresponding to hemagglutinin family protein/adhesin in the 25 µg group and was not observed in the 100 µg group.

### Antigen-specific responses associated with protection following challenge in the H03_03TP study

When analyzing IgG antibody responses at days 57 (pre-challenge) and 85 (29 days post-challenge) in *Shigella*-challenged adults (vaccinated or having received placebo), seven array spots corresponding to five invasive plasmid antigens (IpaB, IpaD, IpaA, IpaH, and IpaC), as well as the invasive plasmid gene C (IpgC) and the outer membrane MxiD protein, showed increased signals. However, only increases in responses to IpaB and IpaH were statistically significant (*P* < 0.05) in participants vaccinated with 1790GAHB. Among participants receiving placebo, increases in responses to IpaB, IpaD, IpaA, IpaH, and IpaC were statistically significant. The average signal at baseline (day 1 in the H03_03TP study) in the vaccine group was comparable to that at day 57 for all array spots (Table 2).

**TABLE 2:** Overview of *Shigella*-specific IgG responses before and after challenge with *S. sonnei* 53G in the H03_03TP study^*a*^^,*b*^

Array spot	Description	Placebo (*N* = 26)			25 µg group (*N* = 32)			
		Average D57	Average D85	Average diff (D85–D57); *P* value	Average D1	Average D57	Average D85	Average diff (D85–D57); *P* value
SF_p0128	IpaB	2.86	4.17	1.31; 0.001	2.33	2.57	3.3	0.73; 0.008
SF_p0126	IpaD	0.85	1.53	0.68; 0.01	1.14	1.00	1.13	0.13; 0.2
SF_p0125	IpaA	0.55	0.99	0.43; 0.03	1.14	1.16	1.20	0.04; 0.4
SF_p0129	IpgC	0.18	0.45	0.27; 0.06	0.51	0.46	0.56	0.10; 0.1
SSON53G_RS27450	IpaH (E3 ubiquitin–protein ligase)	0.98	1.33	0.36; 0.04	1.24	1.24	1.58	0.34; 0.04
SF_p0145	MxiD	−0.12	0.00	0.12; 0.06	0.09	−0.01	0.00	0.01; 0.6
SF_p0127	IpaC	0.79	1.00	0.21; 0.03	1.58	1.43	1.6	0.17; 0.1

^*a*^ D, day; Ipa, invastion plasmid antigen; Ipg, invasion plasmid gene; *N*, number of participants.

^*b*^ Summary statistics of the challenge effect for the placebo and 1790GAHB groups are presented. Array spots with increases in signal of ≥50% from day 57 to day 85 in multiple participants (≥two considering all participants in both groups) are presented and ranked by frequency of increases in signal day 57 to day 85 in the placebo group only. The average signal at day 1 is also given for the 1790GAHB group. *P* values were obtained using a paired *t*-test.

When results were stratified by outcome (shigellosis versus no shigellosis) following challenge, higher pre-challenge IgG levels for array spots corresponding to IpaB, IpaH, and IpaD in the placebo group and IpaB, IpaH, IpaD, IpaC, and IpaA in the 1790GAHB group were associated with a lower risk of shigellosis (*P* < 0.05). Responses to nine proteins in the vaccine group (IpaB, IpaH, IpaD, IpaC, IpaA, MxiD, Spa15, IpgC, and MxiG) and two proteins in the placebo group (IpaB and IpaD) showed an area under the curve higher than 0.70 (Table 3).

**TABLE 3:** Overview of *Shigella*-specific IgG responses before challenge with *S. sonnei* 53G in the H03_03TP study by outcome (shigellosis versus no shigellosis) post-challenge^*a*^^,*b*^

Array spot	Description	Placebo (*N* = 28)				25 µg group (*N* = 32)			
		Average D57		Average diff (Shig− vs. Shig+); *P* value	AUC	Average D57		Average diff (Shig− vs. Shig+); *P* value	AUC
		Shig+ (*N* = 12)	Shig− (*N* = 16)			Shig+ (*N* = 15)	Shig− (*N* = 17)		
SF_p0128	IpaB	1.40	3.97	2.56; 0.0004	0.85	0.67	4.11	3.44; <0.0001	0.92
SSON53G_RS27450	IpaH (E3 ubiquitin–protein ligase)	0.41	1.33	0.92; 0.04	0.65	0.31	1.99	1.68; 0.01	0.81
SF_p0126	IpaD	0.41	1.14	0.73; 0.02	0.72	0.15	1.7	1.55; 0.004	0.87
SF_p0127	IpaC	0.41	0.99	0.58; 0.2	0.58	0.21	2.44	2.23; 0.003	0.80
SF_p0125	IpaA	0.38	0.68	0.30; 0.08	0.66	0.35	1.82	1.47; 0.003	0.89
SF_p0247	oriT	0.2	0.32	0.13; 0.7	0.39	−0.09	0.14	0.23; 0.1	0.62
SF_p0123	VirB	0.08	0.09	0.01; 0.9	0.51	0.05	0.43	0.38; 0.3	0.63
SF_p0145	MxiD	−0.12	−0.11	0.01; 0.9	0.44	−0.19	0.12	0.30; 0.2	0.74
SF_p0146	MxiC	−0.12	−0.13	−0.01; 0.9	0.45	−0.14	0.25	0.39; 0.2	0.55
SF_p0004	PhoN2 (Apy)	−0.36	−0.38	−0.01; 0.9	0.44	−0.32	−0.05	0.27; 0.3	0.61
SF_p0148	Spa15	−0.08	−0.10	−0.02; 0.7	0.45	−0.06	0.52	0.58; 0.2	0.75
SF_p0271	IcsP (SopA)	−0.07	−0.09	−0.03; 0.9	0.41	−0.22	−0.27	−0.05; 0.6	0.54
SF_p0182	IcsA (VirG)	0.12	0.08	−0.04; 0.4	0.42	0.08	0.3	0.22; 0.2	0.62
SF_p0181	VirA	−0.08	−0.13	−0.06; 0.3	0.39	−0.11	0.03	0.14; 0.2	0.61
SF_p0129	IpgC	0.19	0.13	−0.06; 0.6	0.43	0.11	0.73	0.62; 0.07	0.74
SF_p0133	IpgD	0.31	0.23	−0.08; 0.5	0.47	0.3	0.41	0.11; 0.4	0.58
SF_p0136	MxiG	0.54	0.40	−0.13; 0.7	0.45	0.04	0.26	0.22; 0.1	0.73
SF_p0143	MxiM	−0.59	−0.86	−0.27; 0.01	0.20	−0.78	−0.57	0.21; 0.3	0.56
Reactivity count with IpaB excluded		3.33	6.00	2.67; 0.01	0.72	2.13	7.88	5.75; 0.0002	0.90

^*a*^ AUC, area under the curve; D, day; Ipa, invasion plasmid antigen; Ipg, invasion plasmid gene; N, number of participants; Shig+/Shig-, individuals with/without shigellosis following challenge with the *S. sonnei* 53G strain.

^*b*^ Array spots with a normalized signal >1.0 in ≥1 participant at day 57 are presented and are ranked by the average difference between the outcome subgroups considering in the placebo group only. The final row in the table is calculated based on the count of array spots with normalized signals >1.0 on the full set of proteins shown in the table, except IpaB. *P* values were obtained using *t*-test.

Higher pre-challenge IgG responses across all array spots with a normalized signal > 1.0 for ≥1 participant remained associated with a decreased risk of shigellosis when excluding IpaB from the reactive antigen count for each participant (Table 3).

### Microarray baseline reactivity based on varying exposure settings

When comparing across two settings (Kenya and the United States) at baseline (Table 4), there were 47 IVTT array spots with an average normalized intensity at least 0.50 higher in participants from Kenya versus U.S. participants and a *t*-test *P* value < 0.01. This set was reduced by including only one result for each unique protein. Thus, for reactive invasion plasmid proteins, only the *S. sonnei* array spot was kept, and the *S. flexneri* homologue was excluded from the analysis. For large proteins with multiple array spots due to segmenting, only the whole protein array spot was kept in the analysis. Thus, 21 antigens were identified as more reactive (with higher average normalized intensities) in the H03_04TP study (Kenya) than in the H03_03TP trial (U.S.). The first six most reactive proteins (IpaC, IpaB, IpaA, IpaD, IpaH, and IpgC) were also associated with increases in IgG responses following the challenge of U.S. participants in the H03_03TP study (Fig. 3; Table 2). The remaining 15 antigens were highly reactive among a large subset of Kenyan adults (ranging from 19 to 80%) and few of the participants from the United States, except for the collagen triple helix repeat family protein. In the H03_01TP study, no array spot showed high IgG of antibody binding at baseline, except for the spot corresponding to peptidoglycan associated lipoprotein (data not shown).

**TABLE 4:** Overview of baseline microarray reactivity in the H03_04 study in Kenya versus the H03_03 study in the US^*a*^^,*b*^

Array spot	Description	AUC in Kenya; *P* value	Average normalized intensity		Frequency of array spots with normalized signal > 1.0	
			USA	Kenya	USA (*N* = 60)	Kenya (*N* = 46)
SSON53G_RS26830	IpaC	0.97; <0.0001	1.09	6.73	30%	100%
SSON53G_RS26835	IpaB	0.95; <0.0001	2.67	6.71	62%	100%
SF_p0125	IpaA	0.96; <0.0001	0.86	5.64	27%	98%
SSON53G_RS26825	IpaD	0.95; <0.0001	0.91	5.06	28%	98%
SSON53G_RS26690	IpaH	0.98; <0.0001	0.19	4.81	10%	100%
SSON53G_RS26840	IpgC	0.99; <0.0001	0.31	4.23	5%	100%
SSON53G_RS26935	Spa15	0.84; <0.0001	0.09	3.46	3%	79%
SSON53G_RS27105	IcsA (VirG)	0.90; <0.0001	0.15	1.77	2%	77%
Sd_centroid_1550704	Collagen triple helix repeat family protein	0.68; 0.0007	1.07	1.75	38%	69%
SSON53G_RS26810	VirB	0.46; <0.0001	0.17	1.66	2%	40%
SSON53G_RS26310	PhoN2 (Apy)	0.74; <0.0001	−0.27	1.42	2%	58%
SSON53G_RS26920	MxiD	0.86; <0.0001	−0.07	1.40	2%	54%
SSON53G_RS27100	VirA	0.60; <0.0001	−0.07	1.10	2%	38%
SSON53G_RS26925	MxiC	0.40; 0.001	−0.02	1.04	3%	33%
centroid_124167	Patatin-like phospholipase family protein	0.68; 0.0002	0.34	0.93	7%	33%
centroid_779496	yecA family protein	0.58; 0.004	0.18	0.89	7%	29%
SSON53G_RS27490	Chain-length determining protein	0.92; <0.0001	0.06	0.83	2%	35%
SSON53G_RS26320	OspC3	0.54; 0.003	−0.14	0.54	0%	23%
Sb_centroid_2098385	Conserved hypothetical protein	0.70; <0.0001	−0.16	0.47	0%	27%
centroid_2274095	Binding protein-dependent transport system inner membrane component family protein	0.79; <0.0001	−0.07	0.47	0%	23%
SSON53G_RS26910	MxiM	0.77; <0.0001	−0.71	−0.01	2%	19%

^*a*^ AUC, area under the curve; Ipa, invastion plasmid antigen; Ipg, invasion plasmid gene; USA, United States.

^*b*^ The 21 *in vitro* transcription and translation array spots showing higher reactivity among Kenyan participants than U.S. participants are presented and sorted by the average normalized intensity for Kenya participants. *P* values were obtained using *t*-test.

### Correlation between microarray data and *S. sonnei* LPS IgG serum antibody levels

In the H03_01TP study and its extension, the increase from day 1 to post-vaccination timepoints for anti-LPS IgG levels (previously determined [35, 39]) was correlated with the increase in IgG/IgA responses for microarray spots. For IgG responses, the 10 array spots with the highest correlation included four of the five antigens identified as immunogenic following vaccination with 1790GAHB in the H03_01TP and H03_01E1TP studies (Fig. S1A). The highest correlations for IgG responses were identified for T3SS lipochaperone family protein, putative outer membrane lipoprotein Wza, and major outer membrane lipoprotein Lpp. For putative outer membrane lipoprotein Wza, a correlation between IgA microarray data and *S. sonnei* LPS-specific IgG levels was also identified (Fig. S1B).

When using samples from the 25 µg group in study H03_03TP, the 10 array spots with the highest correlation between increases from day 1 to day 57 in IgG protein responses and ELISA data (37) included the putative outer membrane lipoprotein Wza (Fig. S2).

## DISCUSSION

GMMA have been proposed as an innovative delivery system for the *Shigella* OAg (27). They are outer membrane vesicles naturally released from engineered bacteria that resemble the outermost layer of the bacteria and present many protein antigens (31). The *S. sonnei* vaccine candidate 1790GAHB has been tested in different clinical trials, showing an ability to elicit anti-LPS IgG responses and bactericidal activity against the homologous *Shigella* wild-type strain (35–39, 41). In this study, we assessed vaccine-induced *Shigella* protein-specific pan-serotype immune responses using a proteome microarray consisting of 3,150 antigen targets across *Shigella* species.

When considering a threshold of ≥50% increase in IgG responses in the same individual post-vaccination with 1790GAHB, we identified subsets of immunogenic proteins that largely overlapped across studies. In particular, the T3SS lipochaperone family protein, the major outer membrane lipoprotein Lpp, and the putative outer membrane lipoprotein Wza were identified as immunogenic in all studies. The increases in IgG responses to the T3SS lipochaperone family protein were the highest and statistically significant in all three studies. Moreover, the T3SS lipochaperone family protein also induced a strong antibody response following a single vaccination with 1790GAHB. All three proteins are expressed on GMMA (31). Immune responses to these proteins were induced, regardless of the vaccinated population (i.e., naïve or primed participants at baseline), indicating similar response to vaccination with the GMMA-based vaccine across endemic and non-endemic settings. IgG responses to the putative outer membrane lipoprotein Wza also showed a positive correlation with the increased anti-LPS serum IgG titers as measured by ELISA in both the H03_01TP and H03_03TP studies. IgG responses induced by GMMA proteins were dose-dependent, with overall higher values observed in groups receiving 1790GAHB at doses of 5.9/100 compared to 1.5/25 µg of OAg/µg of protein. A similar observation was made for serum anti-LPS IgG responses in participants primed at baseline from the study conducted in Kenya (38). We also observed a cumulative effect of subsequent vaccinations: in the H03_01TP study, where three 1790GAHB doses were administered 4 weeks apart, the highest IgG responses to microarray proteins were observed after the third dose.

We also evaluated anti-protein IgG responses using samples from the CHIM study following challenge with a *S. sonnei* strain. An increase in IgG responses from pre- to post-challenge timepoints was observed for seven T3SS proteins: the invasive plasmid antigens IpaB, IpaD, IpaA, IpaH, and IpaC, the secretin MxiD, and the chaperone IpgC, although only increases in responses to IpaB and IpaH were statistically significant post-challenge, in participants from both vaccinated and placebo groups. IgG immune responses to the same seven proteins pre-challenge were also the highest across the proteome microarray in participants not developing shigellosis. Altogether, these results suggest that IgG responses to these seven proteins may be associated with clinical protection against shigellosis.

The strongest pre-challenge response was observed to IpaB; in line with our findings, a correlation between IpaB-specific IgG and reduced illness was previously identified for experimental challenge with *S. flexneri* 2a as measured by ELISA (23) and when assayed on a *Shigella* proteome microarray consisting of 2,133 antigens (32). When excluding the response to IpaB in our analysis, pre-challenge increased IgG responses to Ipa proteins remained statistically associated with a negative outcome of shigellosis, suggesting that a wider breadth of responses to Ipa proteins can induce protection against shigellosis. None of these proteins are expressed on GMMA (31). IpaB and IpaC are translocators directly interacting with the host plasma membrane, while IpaD makes up the tip complex of the T3S apparatus (42); all have previously been included in several vaccine candidates against *Shigella* (14, 15). IpaA and IpaH have also been identified as potentially immunogenic antigen targets in a *Shigella* proteome microarray experiment with serum samples from adults, whether immunized with *S. flexneri* vaccines or challenged with wild-type *S. flexneri* 2a (32). Here, we also identify, for the first time, MxiD (which makes up the outer ring of the basal body that contacts the needle in the T3SS [43]) and IpgC (a T3SS chaperone protein assisting the folding of IpaB and blocking premature association between IpaB and IpaC [44]) as potentially successful antigens for future vaccine development.

While we have shown that some GMMA-related proteins are immunogenic, with statistically significant increases in IgG response post-vaccination, these increases were not correlated with protection. However, as we only determined IgG and IgA responses to GMMA proteins, we cannot exclude a contribution in terms of antibody functionality that could contribute to protection against disease. Nevertheless, *Shigella* GMMA remain an ideal delivery system for *Shigella* OAg, and because of the low cost of production and high purification yields, they are a particularly attractive vaccine platform for vaccines targeting LMICs (45). While 1790GAHB failed to demonstrate efficacy against shigellosis (37), another GMMA-based *Shigella* vaccine with a higher OAg content has been developed and is currently under evaluation in clinical trials with promising initial results (29).

Moreover, when assaying samples collected at baseline from participants with varying exposure to *Shigella*, we identified a set of 21 *Shigella* antigens, which induced a robust antibody response, higher in Kenya than in the United States, and that was populated mainly with T3SS proteins. The seven most immunogenic proteins in this subset were also associated with increases in IgG responses following challenge with *S. sonnei* 53G in U.S. participants in the H03_03TP CHIM study: IpaC, IpaB, IpaA, IpaD, IpaH, IpgC, and MxiD. This confirms previous findings (14) that T3SS invasive plasmids can be strong candidates as antigens for cross-protective vaccines against *Shigella*. Of note, in participants in the 1790GAHB group in the H03_03TP study, IgG antibody levels against these six proteins (and others) were also detected at baseline. This is further confirmation that part of the participants were not naïve at baseline, although the study was conducted in *Shigella* non-endemic settings. This is in line with our previous findings that 31% of participants in the vaccine group had detectable anti-LPS serum IgG baseline antibody levels (37) that were close to or above levels considered protective in field conditions (46). In contrast, high immune responses were detected against one protein only (peptidoglycan associated lipoprotein) for baseline samples from the H03_01TP study conducted in France, indicating true non-endemic settings.

Our study was limited by the relatively small sample size and the fact that potential natural exposure to *Shigella* had not been monitored throughout the three clinical studies. However, we were able to compare the immune response elicited by GMMA across different populations (i.e., adults with varying exposure to *Shigella*) and compare vaccine-elicited response with anti-protein signatures in naturally exposed individuals.

In conclusion, we identified a set of seven proteins (IpaC, IpaB, IpaA, IpaD, IpaH, IpgC, and MxiD) that were associated with high antibody responses at baseline in endemic populations and correlated with protection from shigellosis after experimental infection, making them a suitable target for future vaccine development. While immunogenic, we found no apparent role of GMMA proteins IgG/IgA responses in protection against disease. Our findings support previous observations that multiple *Shigella* core proteins are relevant for protection and that cross-reactive protective immunity elicited by vaccination may be better achieved with a combination of antigens. Future vaccine development may consider the combination of GMMA as OAg delivery system and potentially protective T3SS proteins as additional antigens.

## Data Availability

Please refer to the GSK URL to access GSK’s data sharing policies and as applicable seek anonymized subject level data: https://www.gsk-studyregister.com/en/.

## SUPPLEMENTAL MATERIAL

Supplemental material msphere.01057-24-s0001.docx Protein microarray construction and supplemental tables and figures.

Table S1 msphere.01057-24-s0002.xlsx *Shigella* pan-proteome microarray: spot identifier, description, and amino acid sequence.

ASM does not own the copyrights to Supplemental Material that may be linked to, or accessed through, an article. The authors have granted ASM a non-exclusive, world-wide license to publish the Supplemental Material files. Please contact the corresponding author directly for reuse.

## References

[1] Centers of Disease Control and Prevention. 2024. CDC Yellow Book 2024 - Shigellosis. Available from: https://wwwnc.cdc.gov/travel/yellowbook/2024/infections-diseases/shigellosis. Accessed 12 June 2024.

[2] Centers of Disease Control and Prevention. 2024. Signs and symptoms of Shigella infection. Available from: https://www.cdc.gov/shigella/signs-symptoms/?CDC_AAref_Val=https://www.cdc.gov/shigella/symptoms.html. Retrieved 14 October 2024.

[3] Khalil IA, Troeger C, Blacker BF, Rao PC, Brown A, Atherly DE, Brewer TG, Engmann CM, Houpt ER, Kang G, et al.. 2018. Morbidity and mortality due to Shigella and enterotoxigenic Escherichia coli diarrhoea: the Global Burden of Disease Study 1990-2016. Lancet Infect Dis 18:1229–1240. doi:10.1016/S1473-3099(18)30475-4 30266330 PMC6202441

[4] GBD 2019 Diseases and Injuries Collaborators. 2020. Global burden of 369 diseases and injuries in 204 countries and territories, 1990-2019: a systematic analysis for the Global Burden of Disease Study 2019. Lancet 396:1204–1222. doi:10.1016/S0140-6736(20)30925-9 33069326 PMC7567026

[5] GBD 2016 Diarrhoeal Disease Collaborators. 2018. Estimates of the global, regional, and national morbidity, mortality, and aetiologies of diarrhoea in 195 countries: a systematic analysis for the Global Burden of Disease Study 2016. Lancet Infect Dis 18:1211–1228. doi:10.1016/S1473-3099(18)30362-1 30243583 PMC6202444

[6] Ranjbar R, Farahani A. 2019. Shigella: antibiotic-resistance mechanisms and new horizons for treatment. Infect Drug Resist 12:3137–3167. doi:10.2147/IDR.S219755 31632102 PMC6789722

[7] Shad AA, Shad WA. 2021. Shigella sonnei: virulence and antibiotic resistance. Arch Microbiol 203:45–58. doi:10.1007/s00203-020-02034-3 32929595 PMC7489455

[8] Shakoor S, Platts-Mills JA, Hasan R. 2019. Antibiotic-resistant enteric infections. Infect Dis Clin North Am 33:1105–1123. doi:10.1016/j.idc.2019.05.007 31668193

[9] Kotloff KL, Riddle MS, Platts-Mills JA, Pavlinac P, Zaidi AKM. 2018. Shigellosis. Lancet 391:801–812. doi:10.1016/S0140-6736(17)33296-8 29254859

[10] Livio S, Strockbine NA, Panchalingam S, Tennant SM, Barry EM, Marohn ME, Antonio M, Hossain A, Mandomando I, Ochieng JB, et al.. 2014. Shigella isolates from the global enteric multicenter study inform vaccine development. Clin Infect Dis 59:933–941. doi:10.1093/cid/ciu468 24958238 PMC4166982

[11] Kotloff KL, Nasrin D, Blackwelder WC, Wu Y, Farag T, Panchalingham S, Sow SO, Sur D, Zaidi AKM, Faruque ASG, et al.. 2019. The incidence, aetiology, and adverse clinical consequences of less severe diarrhoeal episodes among infants and children residing in low-income and middle-income countries: a 12-month case-control study as a follow-on to the global enteric multicenter study (GEMS). Lancet Glob Health 7:e568–e584. doi:10.1016/S2214-109X(19)30076-2 31000128 PMC6484777

[12] Connor TR, Barker CR, Baker KS, Weill F-X, Talukder KA, Smith AM, Baker S, Gouali M, Pham Thanh D, Jahan Azmi I, Dias da Silveira W, Semmler T, Wieler LH, Jenkins C, Cravioto A, Faruque SM, Parkhill J, Wook Kim D, Keddy KH, Thomson NR. 2015. Species-wide whole genome sequencing reveals historical global spread and recent local persistence in Shigella flexneri*.* Elife 4:e07335. doi:10.7554/eLife.07335 26238191 PMC4522646

[13] Thompson CN, Duy PT, Baker S. 2015. The rising dominance of Shigella sonnei: an intercontinental shift in the etiology of bacillary dysentery. PLoS Negl Trop Dis 9:e0003708. doi:10.1371/journal.pntd.0003708 26068698 PMC4466244

[14] Raso MM, Arato V, Gasperini G, Micoli F. 2023. Toward a Shigella vaccine: opportunities and challenges to fight an antimicrobial-resistant pathogen. Int J Mol Sci 24:4649. doi:10.3390/ijms24054649 36902092 PMC10003550

[15] MacLennan CA, Grow S, Ma L, Steele AD. 2022. The Shigella vaccines pipeline. Vaccines (Basel) 10:1376. doi:10.3390/vaccines10091376 36146457 PMC9504713

[16] MacLennan CA, Steele AD. 2022. Frontiers in Shigella vaccine development. Vaccines (Basel) 10:1536. doi:10.3390/vaccines10091536 36146614 PMC9503259

[17] Cohen D, Ashkenazi S, Green MS, Gdalevich M, Robin G, Slepon R, Yavzori M, Orr N, Block C, Ashkenazi I, Shemer J, Taylor DN, Hale TL, Sadoff JC, Pavliakova D, Schneerson R, Robbins JB. 1997. Double-blind vaccine-controlled randomised efficacy trial of an investigational Shigella sonnei conjugate vaccine in young adults. Lancet 349:155–159. doi:10.1016/S0140-6736(96)06255-1 9111538

[18] DuPont HL, Levine MM, Hornick RB, Formal SB. 1989. Inoculum size in shigellosis and implications for expected mode of transmission. J Infect Dis 159:1126–1128. doi:10.1093/infdis/159.6.1126 2656880

[19] Formal SB, Oaks EV, Olsen RE, Wingfield-Eggleston M, Snoy PJ, Cogan JP. 1991. Effect of prior infection with virulent Shigella flexneri 2a on the resistance of monkeys to subsequent infection with Shigella sonnei. J Infect Dis 164:533–537. doi:10.1093/infdis/164.3.533 1869840

[20] Desalegn G, Kapoor N, Pill-Pepe L, Bautista L, Yin L, Ndungo E, Oaks EV, Fairman J, Pasetti MF. 2023. A novel Shigella O-polysaccharide-IpaB conjugate vaccine elicits robust antibody responses and confers protection against multiple Shigella serotypes. mSphere 8:e0001923. doi:10.1128/msphere.00019-23 37017547 PMC10286710

[21] Cohen D, Ashkenazi S, Schneerson R, Farzam N, Bialik A, Meron-Sudai S, Asato V, Goren S, Baran TZ, Muhsen K, Gilbert PB, MacLennan CA. 2023. Threshold protective levels of serum IgG to Shigella lipopolysaccharide: re-analysis of Shigella vaccine trials data. Clin Microbiol Infect 29:366–371. doi:10.1016/j.cmi.2022.10.011 36243351 PMC9993342

[22] de Alwis R, Liang L, Taghavian O, Werner E, The HC, Thu TNH, Duong VT, Davies DH, Felgner PL, Baker S. 2021. The identification of novel immunogenic antigens as potential Shigella vaccine components. Genome Med 13:8. doi:10.1186/s13073-020-00824-4 33451348 PMC7809897

[23] Shimanovich AA, Buskirk AD, Heine SJ, Blackwelder WC, Wahid R, Kotloff KL, Pasetti MF. 2017. Functional and antigen-specific serum antibody levels as correlates of protection against shigellosis in a controlled human challenge study. Clin Vaccine Immunol 24:e00412–e00416. doi:10.1128/CVI.00412-16 27927680 PMC5299116

[24] Bernshtein B, Kelly M, Cizmeci D, Zhiteneva JA, Macvicar R, Kamruzzaman M, Bhuiyan TR, Chowdhury F, Khan AI, Qadri F, Charles RC, Xu P, Kováč P, Clarkson KA, Kaminski RW, Alter G, Ryan ET. 2024. Determinants of immune responses predictive of protection against shigellosis in an endemic zone: a systems analysis of antibody profiles and function. Lancet Microbe 5:100889. doi:10.1016/S2666-5247(24)00112-5 39116906 PMC11488819

[25] Levine MM, Kotloff KL, Barry EM, Pasetti MF, Sztein MB. 2007. Clinical trials of Shigella vaccines: two steps forward and one step back on a long, hard road. Nat Rev Microbiol 5:540–553. doi:10.1038/nrmicro1662 17558427 PMC3771495

[26] Bernshtein B, Ndungo E, Cizmeci D, Xu P, Kováč P, Kelly M, Islam D, Ryan ET, Kotloff KL, Pasetti MF, Alter G. 2022. Systems approach to define humoral correlates of immunity to Shigella. Cell Rep 40:111216. doi:10.1016/j.celrep.2022.111216 35977496 PMC9396529

[27] Micoli F, Adamo R, Nakakana U. 2024. Outer membrane vesicle vaccine platforms. BioDrugs 38:47–59. doi:10.1007/s40259-023-00627-0 37796436 PMC10789842

[28] Micoli F, Nakakana UN, Berlanda Scorza F. 2022. Towards a four-component GMMA-based vaccine against Shigella. Vaccines (Basel) 10:328. doi:10.3390/vaccines10020328 35214786 PMC8880054

[29] Leroux-Roels I, Maes C, Mancini F, Jacobs B, Sarakinou E, Alhatemi A, Joye J, Grappi S, Cilio GL, Serry-Bangura A, et al.. 2024. Safety and immunogenicity of a 4-component generalized modules for membrane antigens Shigella vaccine in healthy European adults: randomized, phase 1/2 study. J Infect Dis 230:e971–e984. doi:10.1093/infdis/jiae273 38853614 PMC11481318

[30] Mancini F, Gasperini G, Rossi O, Aruta MG, Raso MM, Alfini R, Biagini M, Necchi F, Micoli F. 2021. Dissecting the contribution of O-Antigen and proteins to the immunogenicity of Shigella sonnei generalized modules for membrane antigens (GMMA). Sci Rep 11:906. doi:10.1038/s41598-020-80421-y 33441861 PMC7806729

[31] Maggiore L, Yu L, Omasits U, Rossi O, Dougan G, Thomson NR, Saul A, Choudhary JS, Gerke C. 2016. Quantitative proteomic analysis of Shigella flexneri and Shigella sonnei Generalized Modules for Membrane Antigens (GMMA) reveals highly pure preparations. Int J Med Microbiol 306:99–108. doi:10.1016/j.ijmm.2015.12.003 26746581 PMC4820968

[32] Ndungo E, Randall A, Hazen TH, Kania DA, Trappl-Kimmons K, Liang X, Barry EM, Kotloff KL, Chakraborty S, Mani S, Rasko DA, Pasetti MF. 2018. A novel Shigella proteome microarray discriminates targets of human antibody reactivity following oral vaccination and experimental challenge. mSphere 3:e00260-18. doi:10.1128/mSphere.00260-18 30068560 PMC6070737

[33] Tsolakos N, Brookes C, Taylor S, Gorringe A, Tang CM, Feavers IM, Wheeler JX. 2014. Identification of vaccine antigens using integrated proteomic analyses of surface immunogens from serogroup B Neisseria meningitidis. J Proteomics 101:63–76. doi:10.1016/j.jprot.2014.02.013 24561796

[34] Boudová S, Walldorf JA, Bailey JA, Divala T, Mungwira R, Mawindo P, Pablo J, Jasinskas A, Nakajima R, Ouattara A, Adams M, Felgner PL, Plowe CV, Travassos MA, Laufer MK. 2017. Mother-newborn pairs in malawi have similar antibody repertoires to diverse malaria antigens. Clin Vaccine Immunol 24:e00136–00117. doi:10.1128/CVI.00136-17 28835359 PMC5629668

[35] Launay O, Lewis DJM, Anemona A, Loulergue P, Leahy J, Sciré AS, Maugard A, Marchetti E, Zancan S, Huo Z, Rondini S, Marhaba R, Finco O, Martin LB, Auerbach J, Cohen D, Saul A, Gerke C, Podda A. 2017. Safety profile and immunologic responses of a novel vaccine against Shigella sonnei administered intramuscularly, intradermally and intranasally: results from two parallel randomized phase 1 clinical studies in healthy adult volunteers in Europe. EBioMedicine 22:164–172. doi:10.1016/j.ebiom.2017.07.013 28735965 PMC5552227

[36] Micoli F, Rossi O, Conti V, Launay O, Sciré AS, Aruta MG, Nakakana UN, Marchetti E, Rappuoli R, Saul A, Martin LB, Necchi F, Podda A. 2021. Antibodies elicited by the Shigella sonnei GMMA vaccine in adults trigger complement-mediated serum bactericidal activity: results from a phase 1 dose escalation trial followed by a booster extension. Front Immunol 12:671325. doi:10.3389/fimmu.2021.671325 34017343 PMC8129577

[37] Frenck RW Jr, Conti V, Ferruzzi P, Ndiaye AGW, Parker S, McNeal MM, Dickey M, Granada JP, Cilio GL, De Ryck I, Necchi F, Suvarnapunya AE, Rossi O, Acquaviva A, Chandrasekaran L, Clarkson KA, Auerbach J, Marchetti E, Kaminski RW, Micoli F, Rappuoli R, Saul A, Martin LB, Podda A. 2021. Efficacy, safety, and immunogenicity of the Shigella sonnei 1790GAHB GMMA candidate vaccine: results from a phase 2b randomized, placebo-controlled challenge study in adults. EClinicalMedicine 39:101076. doi:10.1016/j.eclinm.2021.101076 34430837 PMC8367798

[38] Obiero CW, Ndiaye AGW, Sciré AS, Kaunyangi BM, Marchetti E, Gone AM, Schütte LD, Riccucci D, Auerbach J, Saul A, Martin LB, Bejon P, Njuguna P, Podda A. 2017. A phase 2a randomized study to evaluate the safety and immunogenicity of the 1790GAHB Generalized Modules for Membrane Antigen vaccine against Shigella sonnei administered intramuscularly to adults from a shigellosis-endemic country. Front Immunol 8:1884. doi:10.3389/fimmu.2017.01884 29375556 PMC5763125

[39] Launay O, Ndiaye AGW, Conti V, Loulergue P, Sciré AS, Landre AM, Ferruzzi P, Nedjaai N, Schütte LD, Auerbach J, Marchetti E, Saul A, Martin LB, Podda A. 2019. Booster vaccination with GVGH Shigella sonnei 1790GAHB GMMA vaccine compared to single vaccination in unvaccinated healthy european adults: results from a phase 1 clinical trial. Front Immunol 10:335. doi:10.3389/fimmu.2019.00335 30906291 PMC6418009

[40] Benjamini Y, Hochberg Y. 1995. Controlling the false discovery rate: a practical and powerful approach to multiple testing. J R Stat Soc Series B Stat Methodol 57:289–300. doi:10.1111/j.2517-6161.1995.tb02031.x

[41] Kapulu MC, Nakakana U, Sciré AS, Sarakinou E, Conti V, Rossi O, Acquaviva A, Necchi F, Obiero CW, Martin LB, Bejon P, Njuguna P, Micoli F, Podda A. 2022. Complement-mediated serum bactericidal activity of antibodies elicited by the Shigella sonnei GMMA vaccine in adults from a shigellosis-endemic country: exploratory analysis of a phase 2a randomized study. Front Immunol 13:971866. doi:10.3389/fimmu.2022.971866 36203568 PMC9531247

[42] Bajunaid W, Haidar-Ahmad N, Kottarampatel AH, Ourida Manigat F, Silué N, Tchagang CF, Tomaro K, Campbell-Valois FX. 2020. The T3SS of Shigella: expression, structure, function, and role in vacuole escape. Microorganisms 8:1933. doi:10.3390/microorganisms8121933 33291504 PMC7762205

[43] Lunelli M, Kamprad A, Bürger J, Mielke T, Spahn CMT, Kolbe M. 2020. Cryo-EM structure of the Shigella type III needle complex. PLoS Pathog 16:e1008263. doi:10.1371/journal.ppat.1008263 32092125 PMC7058355

[44] Ferrari ML, Charova SN, Sansonetti PJ, Mylonas E, Gazi AD. 2021. Structural insights of Shigella translocator IpaB and its chaperone IpgC in solution. Front Cell Infect Microbiol 11:673122. doi:10.3389/fcimb.2021.673122 33996640 PMC8117225

[45] Mancini F, Micoli F, Necchi F, Pizza M, Berlanda Scorza F, Rossi O. 2021. GMMA-based vaccines: the known and the unknown. Front Immunol 12:715393. doi:10.3389/fimmu.2021.715393 34413858 PMC8368434

[46] Cohen D, Green MS, Block C, Rouach T, Ofek I. 1988. Serum antibodies to lipopolysaccharide and natural immunity to shigellosis in an Israeli military population. J Infect Dis 157:1068–1071. doi:10.1093/infdis/157.5.1068 3283258

