# Supplementary material for: Protein-specific immune response elicited by the Shigella sonnei 1790GAHB GMMA-based candidate vaccine in adults with varying exposure to Shigella
Source: mSphere. 2025 Apr 16;10(5):e01057-24. doi: 10.1128/msphere.01057-24 (PMC12108075; doi:10.1128/msphere.01057-24)
Supplement: Supplemental material — Protein microarray construction and supplemental tables and figures. [file msphere.01057-24-s0001.docx]

**Supplemental material**

*Protein-specific immune response elicited by the Shigella sonnei 1790GAHB GMMA-based candidate vaccine in adults with varying exposure to Shigella*

Protein microarray construction

Table S1 (provided as a separate excel file)

Table S2

Table S3

Fig. S1

Fig. S2

**Protein microarray construction**

Fragments were added if the complete gene was over 3,000 base pairs, by splitting the gene into equal segments with an overlap of 500 base pairs. The clone library was created through a high-throughput *in vivo* recombination cloning process (described in detail by Huw Davies et al., doi [10.1073/pnas.0408782102](https://www.pnas.org/doi/full/10.1073/pnas.0408782102)). The polymerase chain reaction (PCR) amplified coding sequences and complementary linearized pXi vector (Antigen Discovery, Inc, Irvine, CA, US) were transformed into competent *Escherichia coli* cells, and recombined plasmids were purified with a Miniprep kit (Qiagen, Germantown, MD, US). Insert sizes were analyzed by agarose gel electrophoresis and quantitative-competitive PCR using gene-specific primers. Open reading frame sequences were confirmed by sequencing (Retrogen Inc., San Diego, CA, US).

Proteins from each clone were expressed using the *E. coli* cell-free rapid translation system (BiotechRabbit, Berlin, Germany). Each expressed protein included a 5’polyhistidine and a 3’ hemagglutinin epitope tag. After expressing the proteins according to the manufacturer’s instructions, translated proteins were printed onto nitrocellulose-coated glass AVID slides (Grace Bio-Labs, Inc., US) using an OmniGrid accent robotic microarray printer (Digilabs, Inc., US). Each slide contained three nitrocellulose pads on which the full array was printed (this allowed three samples to be probed per slide using sealed chambers that isolate the arrays). The printer head consisted of 16 pins arranged in a 4-by-4 grid, which allowed for the printing of 16 array spots (primarily the expressed proteins, but also controls) concurrently, with one spot in each of 16 subarrays. Each set of 16 spots was printed on the three arrays (pads) of the first slide consecutively, then all three arrays on the second slide, and so on for an entire batch of slides.

**Table S2. Average reactivity and variation of raw signals across 24 individual baseline serum samples of endemic participants from the H03_04TP trial, comparing 1:100 and 1:1600 dilutions**

| Protein | CV | | CV delta | Raw average | | Raw average ratio | Normalized average | | Normalized delta |
| --- | --- | --- | --- | --- | --- | --- | --- | --- | --- |
|  | 1:100 | 1:1600 |  | 1:100 | 1:1600 |  | 1:100 | 1:1600 |  |
| IpaB | 0.21 | 0.41 | 0.2 | 192199 | 63163 | 3 | 6.7 | 9.4 | 2.6 |
| IpaH | 0.24 | 0.56 | 0.32 | 167378 | 30575 | 5.5 | 6.5 | 8.2 | 1.7 |
| IpaC | 0.27 | 0.67 | 0.4 | 205054 | 29290 | 7 | 6.8 | 8.1 | 1.3 |
| IpaA | 0.43 | 0.72 | 0.29 | 102517 | 9075 | 11.3 | 5.6 | 6.2 | 0.6 |
| IpaD | 0.5 | 0.79 | 0.29 | 68854 | 6640 | 10.4 | 5 | 5.6 | 0.7 |
| Spa15 | 0.82 | 1 | 0.18 | 41669 | 2803 | 14.9 | 3.7 | 3.9 | 0.1 |
| VirG | 0.61 | 0.9 | 0.29 | 8350 | 495 | 16.9 | 2 | 2.1 | 0.2 |
| IcsB | 2.97 | 3.51 | 0.54 | 7775 | 390 | 19.9 | 0.4 | 0.4 | 0.1 |
| IcsA (VirG) | 0.61 | 0.97 | 0.36 | 6525 | 367 | 17.8 | 1.6 | 1.7 | 0.0 |
| Average of all IVTT spots | 0.86 | 0.33 | -0.53 | 2465 | 139 | 24.3 | 0.0 | 0.0 | 0.0 |

CV, coefficient of variation; IVTT, *in vitro* transcription and translation system.

Note: The proteins shown in the table are all from *S. sonnei* except for IpaA (*S. flexneri*)*.*

**Table S3. Overview of *Shigella*-specific IgG responses after vaccination with 1790GAHB in the H03_01TP study.**

|  | Average difference (D85 vs. D1) | | |  | T-test p-value | | |  | No. of participants with ≥50% increase in signal | | |
| --- | --- | --- | --- | --- | --- | --- | --- | --- | --- | --- | --- |
| Array spot | 25 µg | 100 µg | Placebo |  | 25 µg | 100 µg | Placebo |  | 25 µg | 100 µg | Placebo |
| centroid_1428508 | 2.33 | 2.09 | -0.03 |  | 0.01 | 0.0007 | 0.3 |  | 5 | 8 | 0 |
| centroid_1452641 | 0.40 | 0.97 | -0.13 |  | 0.3 | 0.003 | 0.6 |  | 3 | 6 | 0 |
| centroid_282246 | 1.14 | 2.06 | 0.03 |  | 0.05 | 0.004 | 0.4 |  | 3 | 6 | 0 |
| centroid_1637890 | 0.21 | 1.08 | -0.11 |  | 0.2 | 0.007 | 0.02 |  | 1 | 7 | 0 |
| centroid_274565 | 0.46 | 0.76 | -0.02 |  | 0.02 | 0.003 | 0.7 |  | 3 | 5 | 0 |
| centroid_1306389 | 0.18 | 1.11 | 0.04 |  | 0.1 | 0.005 | 0.6 |  | 1 | 6 | 0 |
| centroid_476366 | 0.35 | 0.82 | -0.04 |  | 0.02 | 0.01 | 0.5 |  | 2 | 5 | 0 |
| centroid_601829 | 0.53 | 0.60 | -0.04 |  | 0.02 | 0.0002 | 0.5 |  | 2 | 4 | 0 |
| centroid_223856 | 0.38 | 0.41 | 0.12 |  | 0.2 | 0.03 | 0.2 |  | 1 | 4 | 0 |
| centroid_468404 | 0.26 | 0.62 | -0.01 |  | 0.00007 | 0.006 | 0.8 |  | 0 | 5 | 0 |
| Sd_centroid_11661 | 0.22 | 0.60 | -0.08 |  | 0.07 | 0.004 | 0.4 |  | 1 | 4 | 0 |
| centroid_1279199 | 0.03 | 0.73 | 0.01 |  | 0.3 | 0.04 | 0.8 |  | 0 | 4 | 0 |
| Whole array | 0.01 | 0.04 | -0.04 |  |  |  |  |  | 31 | 92 | 3 |

D, day.

Note: The array spots shown are the ones on the array where participants had an increase in signal from day 1 to day 85 of ≥50%. Array spots are ordered by the combined count of participants with ≥50% increase in signal. Results for the whole array are also shown.

**Fig. S1. Scatterplots of the increase in normalized intensity of signals for individual array spots and the base-2 log of the *S. sonnei* LPS-specific IgG (A) and IgA (B) levels as determined by ELISA for the 10 antigens with the highest Spearman correlations in studies H03_01TP and H03_01E1TP.** The Spearman correlation coefficient and p-value are given in red font for each scatterplot.

.
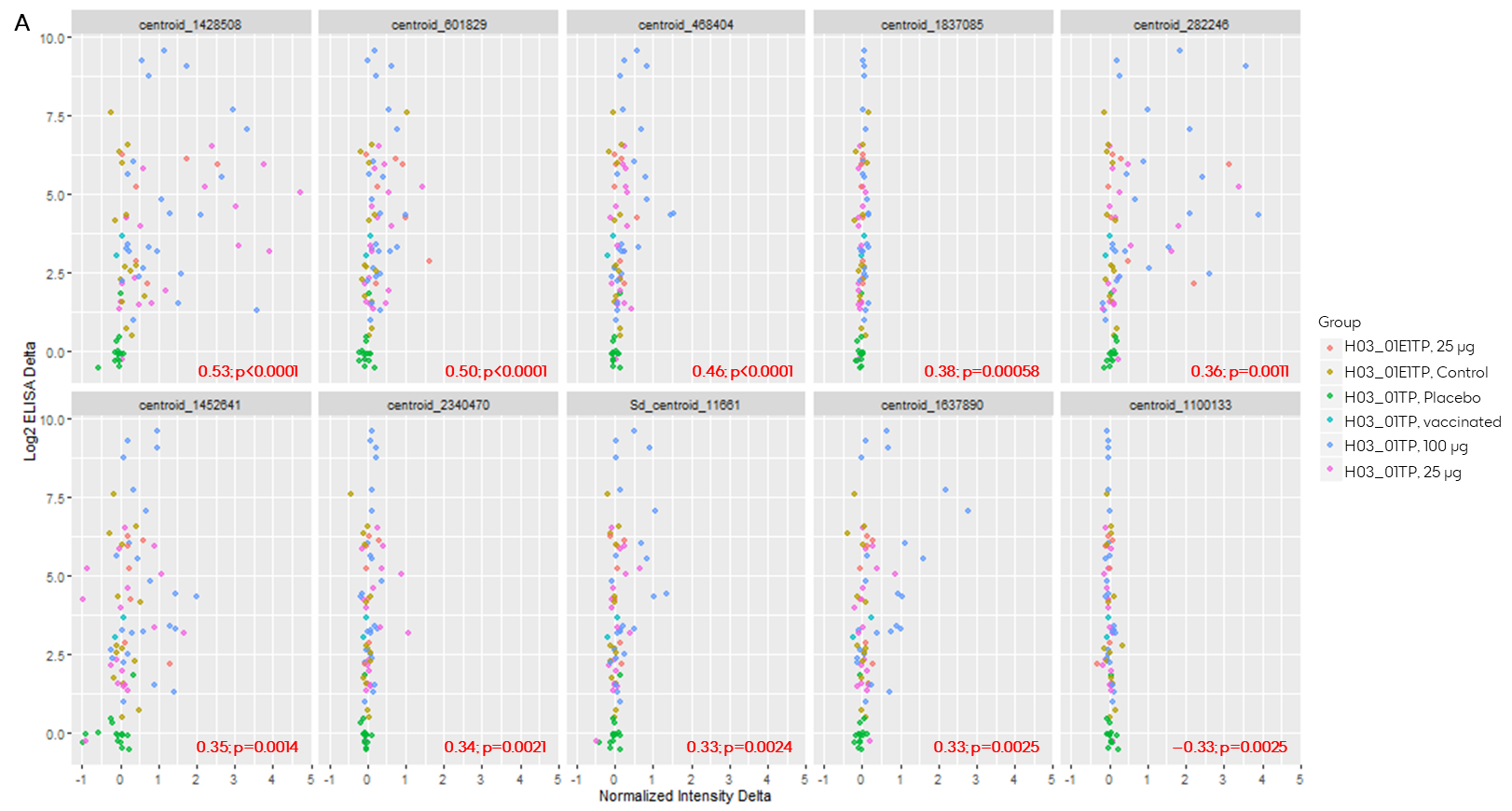


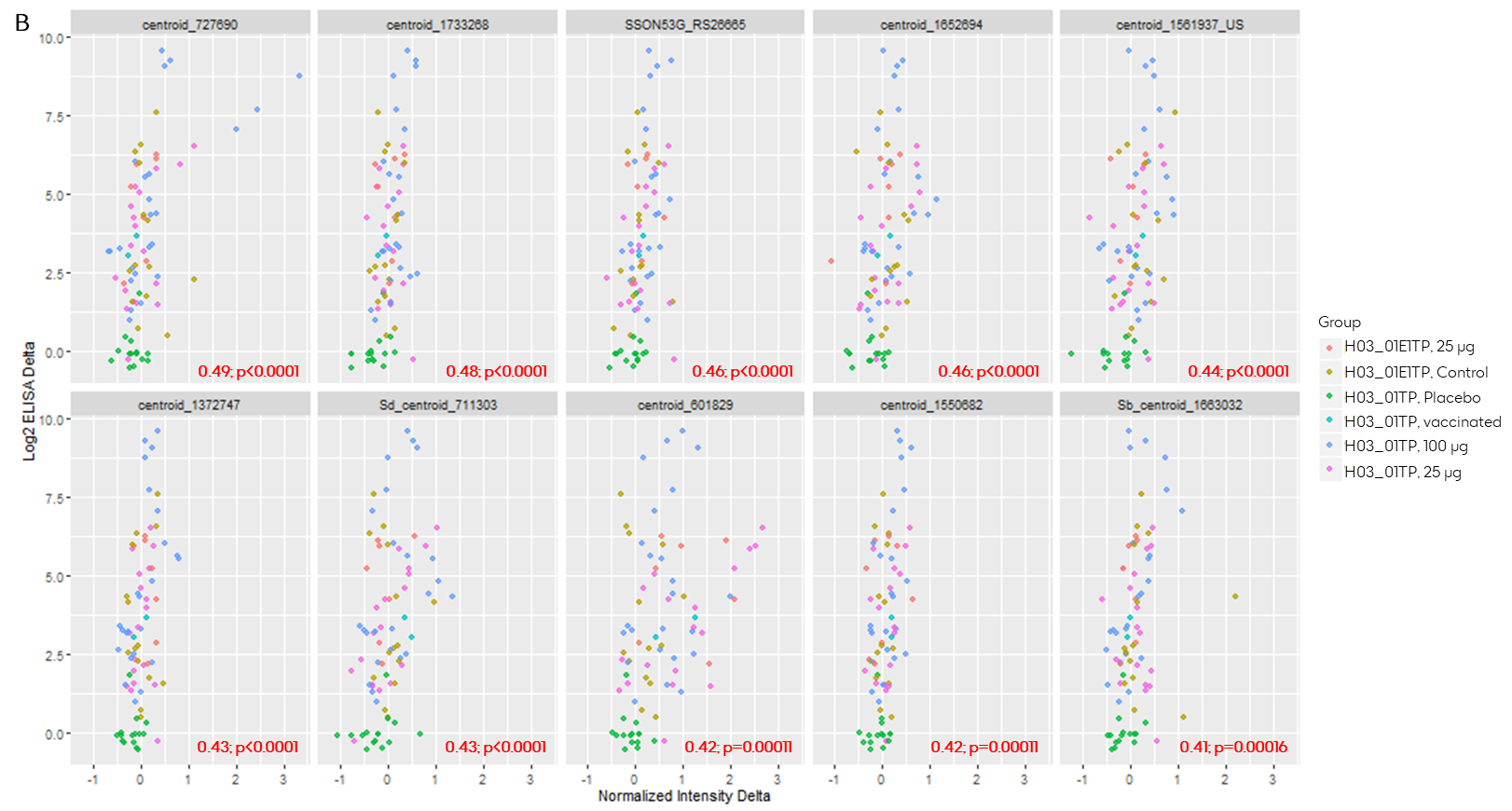


LPS, lipopolysaccharide; Ig, immunoglobulin; ELISA, enzyme linked immunosorbent assay.

**Fig. S2. Scatterplots of the increase in normalized intensity of signals for individual array spots and the base-2 log of the *S. sonnei* LPS-specific IgG (levels as determined by ELISA for the 10 antigens with the highest Spearman correlations in study H03_03TP).** The Spearman correlation coefficient and p-value are given in red font for each scatterplot.


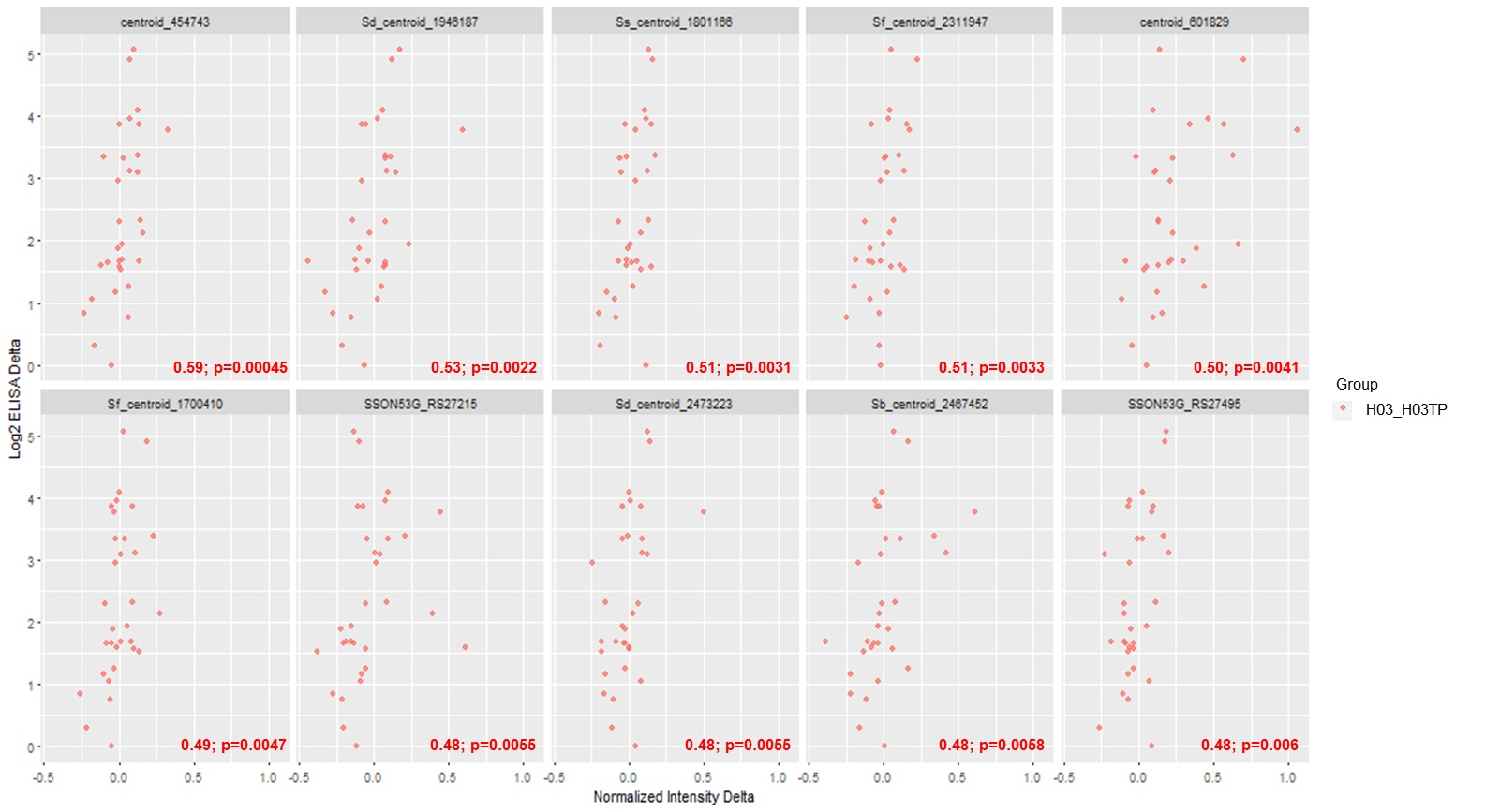


LPS, lipopolysaccharide; Ig, immunoglobulin; ELISA, enzyme linked immunosorbent assay.
